# Biolistic-delivery-based transient CRISPR/Cas9 expression enables *in planta* genome editing in wheat

**DOI:** 10.1038/s41598-018-32714-6

**Published:** 2018-09-26

**Authors:** Haruyasu Hamada, Yuelin Liu, Yozo Nagira, Ryuji Miki, Naoaki Taoka, Ryozo Imai

**Affiliations:** 1 Division of Applied Genetics, Institute of Agrobiological Sciences, National Agriculture and Food Research Organization, 2-1-2 Kannondai, Tsukuba, 305-8602 Japan; 2 Biotechnology Research Laboratories, KANEKA CORPORATION, Takasago, Japan

## Abstract

The current application of genome editing to crop plants is limited to cultivars that are amenable to *in vitro* culture and regeneration. Here, we report an *in planta* genome-editing which does not require callus culture and regeneration. Shoot apical meristems (SAMs) contain a subepidermal cell layer, L2, from which germ cells later develop during floral organogenesis. The biolistic delivery of gold particles coated with plasmids expressing CRISPR/Cas9 components designed to target *TaGASR7* were bombarded into SAM-exposed embryos of imbibed seeds. Bombarded embryos showing transient GFP expression within SAM were selected and grown into adult plants. Mutations in the target gene were assessed in fifth-leaf tissue by cleaved amplified polymorphic sequence analysis. Eleven (5.2%) of the 210 bombarded plants carried mutant alleles, and the mutations of three (1.4%) of these were inherited in the next generation. Genotype analysis of T_1_ plants identified plants homozygous for the three homeologous genes, which were all derived from one T_0_ plant. These plants showed no detectable integration of the Cas9 and guide RNA genes, indicating that transient expression of CRISPR/Cas9 introduced the mutations. Together, our current method can be used to achieve *in planta* genome editing in wheat using CRISPR/Cas9 and suggests possible applications to other recalcitrant plant species and variations.

## Introduction

Genome editing has been successfully applied in major crops, such as rice (*Oryza sativa* L.), maize (*Zea mays* L.) and wheat (*Triticum aestivum* L.), using clustered regularly interspaced short palindromic repeats (CRISPR) and CRISPR-associated protein9 (Cas9) nuclease, which is a simple and versatile tool for inducing DNA double-stranded breaks at target DNA sites^1–3^. In these cases, CRISPR/Cas9 expression cassettes as well as a selectable marker gene were introduced into plant genomes using *Agrobacterium tumefaciens*-mediated or biolistic delivery. Recently, DNA-free genome editing has been achieved in several crops, including lettuce (*Lactuca sativa* L.), maize and wheat, with a mixture of Cas9 mRNA and guide RNA or pre-assembled ribonucleoproteins (RNPs) directly introduced into the cells^4–6^. However, current applications of the CRISPR/Cas9 system in plants rely on the conventional plant transformation procedure that includes callus culture and regeneration processes. This limits application of this technology to cultivars that are amenable to tissue culture. Many elite commercial cultivars lack this property and thus are recalcitrant to transformation. In addition, the callus culture process is generally time-consuming and can suffer from somatic variation.

To avoid the problems associated with the callus culture and regeneration processes during plant transformation, an *in planta* method, in which transgenes are introduced directly into intact immature florets through *Agrobacterium*, has been developed in *Arabidopsis thaliana*. *In planta* transformation procedures have also been reported in several crops, such as rice, maize, tomato and wheat^7–10^. However, these methods seem to suffer from problems with reproducibility and efficiency and are not established as standard protocols. Recently, an alternative method using biolistic delivery, *in planta* particle bombardment (iPB), has been developed in wheat^11^. The iPB method utilizes shoot apical meristem (SAM) of imbibed seeds as the target cells for transformation. With this method, it is now possible to transform wheat cultivars that are recalcitrant to conventional transformation procedures.

Here, we report an *in planta* genome-editing procedure in wheat using iPB. We show that transient expression of CRISPR/Cas9 genes within the SAM of imbibed seed embryos induces genome editing and the plants grown from the embryos inherit the edited sequence to the next generation. The method can be used to introduce DNA-free genome-editing in wheat.

## Results

### Detection of CRISPR/Cas9-mediated genome editing in SAM

**Figure 1:**
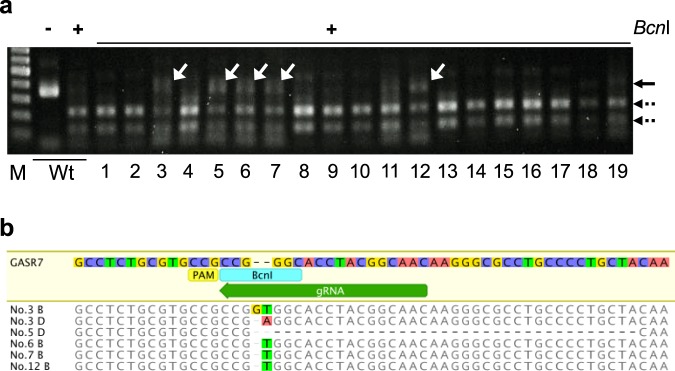
CAPS analysis of *TaGASR7* locus in meristematic tissue. **(a)** Genomic DNA was isolated from the meristematic tissue of wild-type (Wt) and GFP-positive embryos (nos. 1–19) 3 days after plant bombardment and then subjected to a cleaved amplified polymorphic sequences (CAPS) assay. M, marker; −, undigested PCR products; + , *Bcn*I-digested PCR products. Solid and dashed arrows indicate the positions of uncut and cut PCR products, respectively. The full-length gel image is shown in Supplementary Fig. S2. **(b)** DNA extracted from the undigested bands (nos. 3, 5, 6, 7 and 12 in (a)) indicated by white arrowheads were randomly sequenced. The guide sequence is indicated by a green arrow. The protospacer-adjacent motif (PAM) sequence and *Bcn*I restriction site are indicated by yellow and blue bars, respectively.

To achieve *in planta* genome-editing in wheat, we selected *TaGASR7*, which is involved in the control of grain length and weight and has been shown to be amenable to genome editing with CRISPR/Cas9, as the target gene^12,13^. We designed a single guide RNA (sgRNA) targeting all three homeologous *TaGASR7* genes (*TaGASR7-A1*, *-B1* and *-D1*) according to the previous report^12^. We mixed three plasmid constructs carrying expression cassettes for *Staphylococcus pyogenes* Cas9, the sgRNA and a *GFP* reporter gene, respectively, coated gold particles with the mixture, and then bombarded shoot apical meristems (SAMs) of mature embryos with the coated gold particles according to the standard iPB delivery protocol^11^. We observed the bombarded SAMs under a fluorescence microscope to check for transient GFP expression (Supplementary Fig. S1a,b). We defined embryos showing one or more GFP spots within the SAM (19 of the 30 bombarded) as GFP positive and selected these for further study (Supplementary Fig. S1c,d). No wound-induced auto-fluorescence was observed in the bombarded SAMs^11^. Three days after the bombardment, we excised the SAMs of the embryos to test for targeted mutagenesis of the *TaGASR7* genes. Cleaved amplified polymorphic sequences (CAPS) analysis revealed that five embryos (nos. 3, 5, 6, 7 and 12) showed undigested bands after *Bcn*I digestion, suggesting that mutations had occurred at the *Bcn*I target site (Fig. 1a). Subsequent small-scale sequence analysis of the undigested bands revealed several patterns of mutations in *TaGASR7* genes (Fig. 1b). These results suggested that Cas9 and sgRNA expression cassettes were successfully delivered into the meristematic region and caused targeted mutagenesis within 3 days.

### Confirmation of targeted mutagenesis in T_0_ progeny and T_1_ progeny

**Figure 2:**
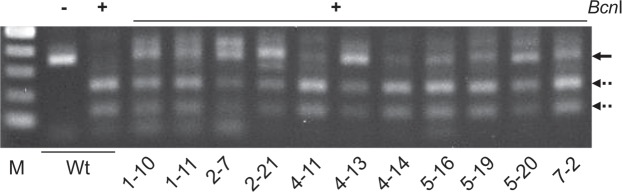
CAPS analysis of *TaGASR7* locus in T_0_ plants. Genomic DNA was isolated from each fifth leaf of 11 bombarded plants and one wild-type (Wt) plant and then subjected to PCR and subsequent *Bcn*I restriction enzyme digestion. M, marker; −, undigested PCR products; + , *Bcn*I-digested PCR products. Black and dashed arrowheads indicate the positions of uncut and cut PCR products, respectively. The full-length gel image is shown in Supplementary Fig. S3.

**Figure 3:**
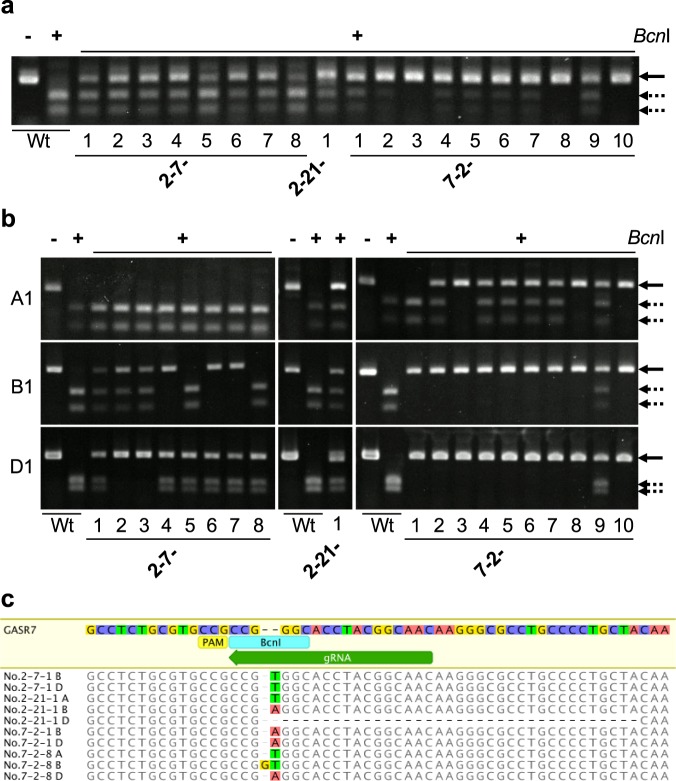
CAPS analysis of the *TaGASR7-A1*, *-B1* and *-D1* loci in T_1_ plants. Genomic DNA isolated from each first leaf of independent T_1_ plants derived from three T_0_ mutants (2-7, 2-21, and 7-2) and one wild-type (Wt) plant. The DNA was subjected to PCR with *TaGASR7*-*A1, -B1* and *-D1* conserved **(a)** and specific **(b)** primer sets. PCR products were digested with *Bcn*I restriction enzyme. −, undigested PCR products; + , *Bcn*I-digested PCR products. Solid and dashed arrows indicate the positions of uncut and cut PCR products, respectively. The full-length gel image of Fig. 3(a) is shown in Supplementary Fig. S4. **(c)** Mutant alleles identified in T_1_ mutant plants. The guide sequence is highlighted by a green arrow. The PAM sequence and *Bcn*I restriction site are indicated by yellow and blue bars, respectively.

Next, we conducted a larger-scale screening of genome-edited plants in T_0_ progeny. Of the 210 bombarded embryos, we selected 176 (83.8 ± 4.3%, *n* = 7) that transiently expressed GFP in their meristematic regions and grew these into mature plants (Table 1). We tested the targeted mutagenesis of the *TaGASR7* genes in the fifth leaf of each plant using a CAPS assay. We detected undigested bands in 11 plants, accounting for 5.2% of the bombarded embryos (Fig. 2, Table 1), which implies that targeted mutations in the meristematic region can be reflected in the genotype of the young leaf of T_0_ plants. We grew the 11 selected plants to obtain seeds and then, because of the possible chimeric nature of the T_0_ plants, subjected all of these T_1_ seeds to genotype analysis. The CAPS assay detected mutant alleles of *TaGASR7* genes in one or more T_1_ plants (2-7-1 to 2-7-8, 2-21-1, and 7-2-1 to 7-2-10) derived from 3 of the 11 putative T_0_ mutant plants (2-7, 2-21 and 7-2) (Fig. 3a, Tables 1, 2). T_1_ plants derived from the 2–7 T_0_ plant contained mutations in the B and D genomes but not the A genome. The 2–21 T_0_ plant set five T_1_ seeds, of which one had heterozygous mutations in the A, B and D genomes (Table 2). Three T_1_ plants (7-2-3, 7-2-8 and 7-2-10) from the 7–2 T_0_ plant carried homozygous mutations in all three genomes (Fig. 3b, Table 2). Sequencing analysis of the *Bcn*I-resistant PCR amplicons revealed either a T, A or GT insertion or a 35-bp deletion in one or more of the *TaGASR7* genes in each of these T_1_ plants (Fig. 3c, Supplementary Table 1). The 7–2–8 plant showed insertions of T, GT and A into the *TaGASR7* genes of the A, B and D genomes, respectively, and thus was considered to carry a complete knockout of *TaGASR7* (*Tagasr7*). The inheritance of the mutations in T_1_ plants suggested that the Cas9 and sgRNA cassettes are efficiently introduced into meristematic L2 cells and cause mutations in germline cell(s).

**Table 1:** Summary of genome editing experiment using the iPB method.

Target gene	Bulk no.	No. of bombarded plants	No. of plants with GFP expression in SAM* (%)	No. of mutants in T_0_ progeny** (%)	No. of mutants in T_1_ progeny** (%)
*TaGASR7*	1	30	26	2	0
	2	30	29	2	2
	3	30	27	0	0
	4	30	22	3	0
	5	30	29	3	0
	6	30	22	0	0
	7	30	21	1	1
total		210	176 (83.8)	11 (5.2)	3 (1.4)

^*^Plants with one or more fluorescent GFP spots within SAM were considered GFP positive.

^**^Mutants were identified through CAPS and sequence analyses in the fifth leaf of T_0_ progeny or the first leaf of T_1_ progeny.

**Table 2:** Number of genome-edited plants in T_1_ progeny.

ID no. of T_0_ plants	Spike	Total no. of T_1_ seeds	Wild-type plants	Plants with mutated alleles*	Plants with biallelic mutations**
2-7	Subsidiary	23	15	8	0
2-21	Primary	5	4	1	0
7-2	Subsidiary	10	0	7	3

^*^Wheat plants carrying one or more mutant alleles were considered mutants.

^**^Wheat plants carrying all mutant alleles were considered mutants with biallelic mutations.

### Detection of CRISPR/Cas9 vector DNA sequences

**Figure 4:**
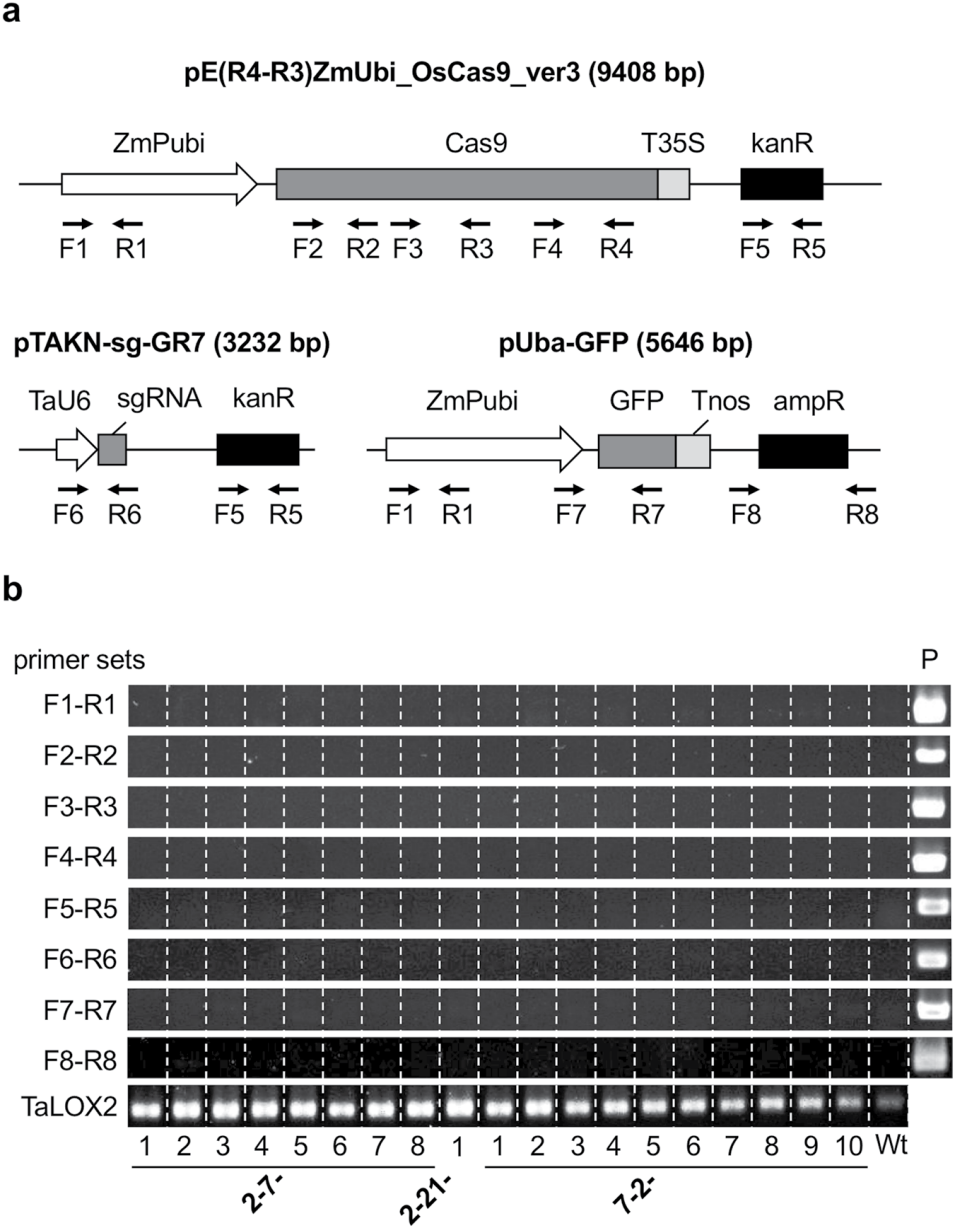
Detection of foreign DNA integration in T_1_ mutant lines. **(a)** Schematic structure of the bombarded plasmids and primer sets used to detect DNA integration. ZmPubi, maize *ubiquitin* promoter; T35S, cauliflower mosaic virus 35S terminator; kanR, kanamycin-resistance gene; TaU6, wheat *U6* promoter; ampR, ampicillin-resistance gene. **(b)** Genomic PCR analysis of T_1_ mutants (2-7-1 to 2-7-8, 2-21-1, and 7-2-1 to 7-2-10) and wild-type (Wt) plants. Genomic DNA was extracted from the first leaf of each T_1_ plant. The corresponding vector DNA was used as a positive control (P). The full-length gel image is shown in Supplementary Fig. S5.

Genes introduced into plant cells via particle bombardment can be expressed transiently without genome integration. This suggested that genome editing using iPB can be achieved by transient expression of the CRISPR/Cas9 system without stable chromosomal integration of the genes required for CRISPR/Cas9. To test this possibility, we carried out genomic PCR to detect the possible integration of the introduced plasmids. We designed primer sets to detect eight distinct DNA regions within the expression plasmids pE(R4-R3)ZmUbi_OsCas9_ver3, pTAKN-sg-GR7 and pUba-GFP (Fig. 4a, Supplementary Table 2) and analysed genomic DNA from all 19 T_1_ plants (2-7-1 to -8, 2-21-1, and 7-2-1 to -10). None of the T_1_ mutants, including the three homozygous mutants, showed an amplification signal for any of the eight DNA regions that was comparable in level to that of *TaLOX2*, which is a single-copy gene in wheat genomes (Fig. 4b, Supplementary Table 1). These results indicated that none of the genome-edited plants contained a functional copy of the CRISPR/Cas9 genes, suggesting that the mutations were created through transient expression of CRISPR/Cas9 in SAM.

## Discussion

Genome editing has been applied to major crops such as rice, maize, wheat and potato^1–3,12,14^. The current strategy for editing genes in such crops depends on tissue culture, and this limits application of genome editing to the specific cultivars amenable to callus culture and regeneration. In this study, we report an *in planta* method for genome editing in wheat that does not require callus culture and regeneration.

We utilized meristematic tissue of the mature embryo as the target tissue for bombardment. As indicated by *GFP* expression analysis (Supplementary Fig. S1, Table 1), gold particles can be efficiently delivered to the SAM region of the embryos. Analysis of genomic DNA isolated from SAM of the bombarded embryos indicated that genome editing occurred in a high proportion of the embryos within 3 days after bombardment (Fig. 1). These data suggested that genome editing might occur without integration and stable expression of CRISPR/Cas9 genes. This was confirmed by PCR analysis of T_1_ plants (Fig. 4). It is interesting to note that the pattern of mutations detected in the embryos was similar to that detected in T_1_ plants (Figs 1b and 3c), even though these mutations were created independently. Clearly, there is a preference for the base modification during DNA repair. The preference may be varied by cultivar and experimental system utilized^12^.

The iPB method is designed to target particle delivery of CRISPR/Cas9 expression cassettes to SAM. In imbibed seeds, the first three leaves have already developed. The fourth and later leaves appear after this point and therefore can show phenotypic evidence of mutations at this stage. We therefore performed our first selection of the genome-edited plants with fifth-leaf tissue, and we found that about 5% of T_0_ plants showed detectable mutations (Fig. 2, Table 1). Within the SAM, L2 cells are the only source of germ cells. Therefore, heritable mutations should be introduced into the L2 cells. In total, 1.4% of the T_0_ plants produced T_1_ seeds with inherited mutations (Table 1). This suggested that a fairly high proportion of mutations can be induced in L2 cells under the conditions used in this study.

Besides its potential application to commercial cultivars, the iPB method has several merits over conventional genome-editing methods. i) Experiments can be started quickly and easily with dry mature seeds; it is not necessary to prepare immature embryos. ii) No antibiotic selection is necessary. iii) In addition to DNA, RNA and protein can also be introduced into plant cells with gold particles; CRISPR/Cas9 RNP has been successfully delivered into maize and wheat cells by this method to induce genome editing^5,6^.

DNA-free genome editing is favoured for commercial applications of genome-edited crops, because it may reduce the chance of off-target changes and it alleviates concerns about genetically modified organisms^12^. The iPB method with CRISPR/Cas9 DNA successfully created genome-edited wheat plants through transient expression of the CRISPR-Cas9 genes. However, it is still possible that a small fragment of the plasmids might be integrated into the chromosomes of the mutants. To avoid this possibility, the next avenue of exploration will be the use of CRISPR/Cas9 RNP instead of DNA. In any event, we believe the iPB method will be a useful tool for genome editing in a wide range of wheat cultivars.

## Materials and Methods

### Preparation of mature embryos

Preparation of mature embryos was performed as previously reported^11^. In brief, mature seeds of wheat (*Triticum aestivum* L. cv. Bobwhite) were sterilised and rinsed and then germinated overnight at 22 °C. The parts of the coleoptile and leaf primordia covering the SAM were excised. The embryos were separated from endosperms and placed upright on Murashige and Skoog (MS) basal medium supplemented with plant preservative mixture (3%; Nacalai Tesque, Japan) in culture plates. Thirty embryos per plate were placed in a circle (diameter 0.8 cm).

### Expression vector

The wheat U6 promoter and gRNA scaffold^15^ were synthesised *de novo* and cloned into the plasmid pTAKN-2 by TA cloning. The guide sequence^12^ for the *TaGASR7-A1*, *-B1*, and *-D1* genes were inserted between the two *Bbs*I sites of the plasmid. The resulting vector (pTAKN-sg-GR7) was used for expression of sgRNA. Wheat SAMs were bombarded with pE(R4-R3)ZmUbi_OsCas9_ver3, pTAKN-sg-GR7 and pUba-GFP^11^.

### Preparation of microprojectiles and biolistic delivery

Microprojectiles were prepared as previously reported with slight modifications^11^. Briefly, pE(R4-R3)ZmUbi_OsCas9_ver3 (5 μg), pTAKN-sg-GR7 (3 μg) and pUba-GFP (2 μg) were mixed with gold particles (InBio Gold, Australia) of 0.6 μm. Bombardment was conducted using a PDS-1000/He™ device (Bio-Rad, USA) with a target distance of 6.0 cm from the stopping plate. The vacuum in the chamber was 27 inches of Hg and the helium pressure was 1350 psi. Bombardment was repeated four times per plate.

### Microscopic analyses and plant growth conditions

About 12 h after bombardment, SAMs in the bombarded mature embryo were observed with an MZFLIII microscope equipped with a GFP filter (excitation wavelength, 470/40 nm; emission wavelength, 525/50 nm). Mature embryos expressing GFP in the SAMs were transferred into a Phytatray™ II (Sigma-Aldrich, USA) with basal MS medium and cultivated for 2–3 weeks in a growth chamber under long day conditions (16 h light/8 h darkness) at 22 °C. Seedlings were planted in pots (3 seedlings/pot, φ 10.5 cm) and grown in a phytotron under long day conditions at 22 °C.

### Detection of vector DNA sequences

To detect vector DNA sequences in T_1_ mutants, polymerase chain reaction (PCR) analysis was conducted. DNA was isolated from the first leaf of the T_1_ progeny, as described previously^16^. Each round of PCR was conducted in a reaction mixture (10 µL) containing dNTP (0.2 mM of each), 1 × PrimeSTAR GXL Buffer, primer (300 nM of each), PrimeSTAR GXL DNA Polymerase (0.25 U; TaKaRa, Japan) and genomic DNA (15 ng). The mixture was denatured (for 2 min at 98 °C) in a thermocycler and then subjected to 32 cycles of amplification (98 °C for 10 sec, 60 °C for 15 sec, and 68 °C for 30 sec). PCR amplification to select DNA-integrated plants was performed with the primers described in Supplementary Table 2. A lipoxygenase gene (*LOX2*) was analysed as a quantitative control. Half of each PCR product was resolved by agarose gel electrophoresis and visualised by staining with ethidium bromide under UV light.

### Cleaved amplified polymorphic sequences (CAPS)

Meristematic tissue was dissected from an embryo 3 days after bombardment. DNA was isolated from the meristematic tissue using 2 µL DNAzol Direct (Cosmo Bio, Japan). DNA from the fifth leaf (of the T_0_ progeny) and the first leaf (of the T_1_ progeny) was isolated as described previously^16^. Each round of PCR was conducted in a reaction mixture (10 µL) containing dNTP (0.2 mM of each), 1 × PCR Buffer for KOD FX Neo, primers (300 nM of each), KOD FX Neo (0.20 U; TOYOBO, Japan) and genomic DNA (0.3 µL for meristematic tissue or 15 ng for leaf tissue). The mixture was denatured for 2 min at 94 °C in a thermocycler and then subjected to 32 cycles for leaf tissue or 35 cycles for meristematic tissue of amplification (98 °C for 10 sec, 68 °C for 30 sec). *TaGASR7* genes conserved or specific primer sets were described in Supplementary Table 2. The specific primer sets were designed according to the previous report^12^. PCR products were digested with the restriction enzyme *Bcn*I and then analysed by agarose gel electrophoresis. The purified PCR products from the enzyme-durable bands were subjected to further sequencing analysis.

### Sequencing analysis

PCR products used in the CAPS analysis were cloned into pCR-BluntII-TOPO (Thermo Fisher Scientific, USA) and sequenced with a 3130xL genetic analyser (Applied Biosystems, USA).

## Electronic supplementary material

Supplementary information

## Data Availability

All data generated or analysed during this study are included in this published article (and its Supplementary Information files).
