# Supplementary material for: Biolistic-delivery-based transient CRISPR/Cas9 expression enables in planta genome editing in wheat
Source: Sci Rep. 2018 Sep 26;8:14422. doi: 10.1038/s41598-018-32714-6 (PMC6158239; doi:10.1038/s41598-018-32714-6)
Supplement: Supplementary file 1 — Supplementary information [file 41598_2018_32714_MOESM1_ESM.pdf]

Supplementary Information

Biolistic-delivery-based transient CRISPR/Cas9 expression enables *in planta* genome editing in wheat

Haruyasu Hamada[2], Yuelin Liu[1], Yozo Nagira[2], Ryuji Miki[2], Naoaki Taoka[2] & Ryozo Imai[1*]

[1] Division of Applied Genetics, Institute of Agrobiological Sciences, National Agriculture and Food Research Organization, 2-1-2 Kannondai, Tsukuba 305-8602, Japan

[2]Biotechnology Research Laboratories, KANEKA CORPORATION, Takasago, Japan.

*Correspondence and requests for materials should be addressed to R.I. (e-mail: rzi@affrc.go.jp).

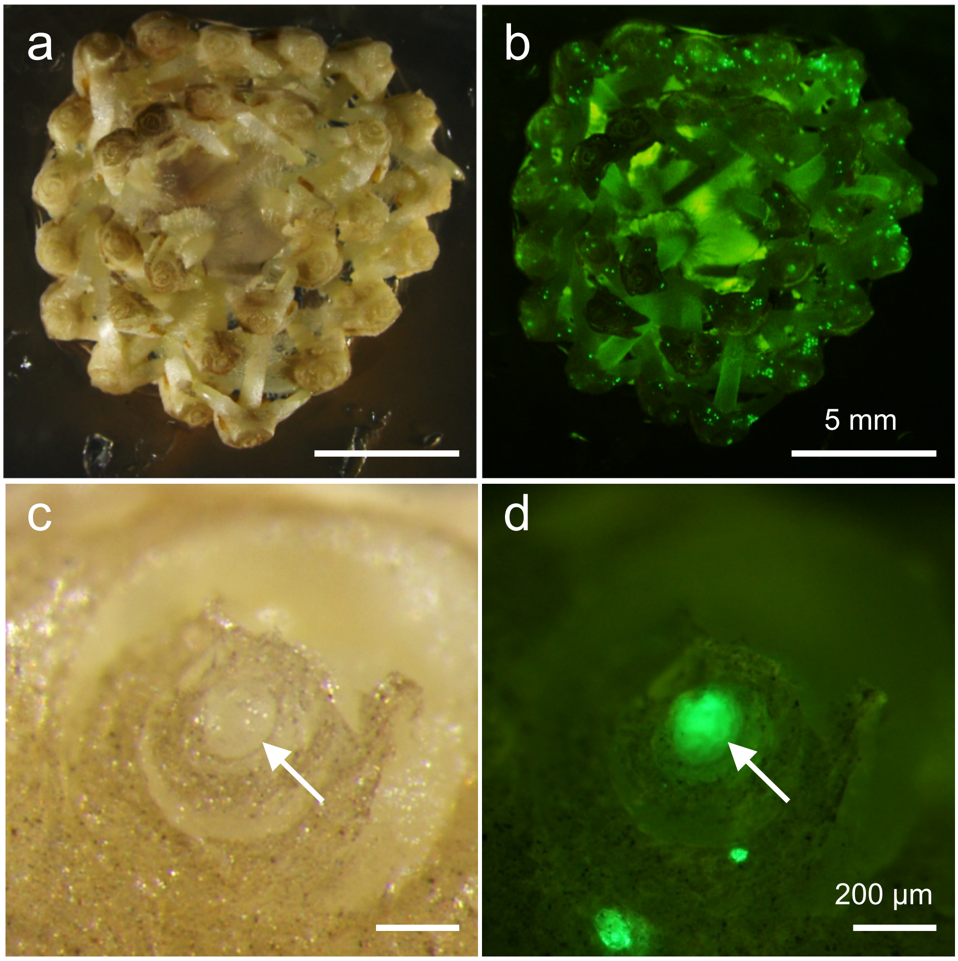

Supplementary Figure S1. Transient GFP expression in shoot apical meristems.

Wheat shoot apical meristems (SAMs) were bombarded with plasmid constructs for CRISPR/Cas9 and GFP expression using 0.6 µm particles and 1,350 psi helium pressure. **(a)** Bright field and **(b)** fluorescence images of whole apical tissues 12 h after bombardment. **(c)** Bright field and **(d)** fluorescence images of a SAM; SAMs are indicated by arrows.

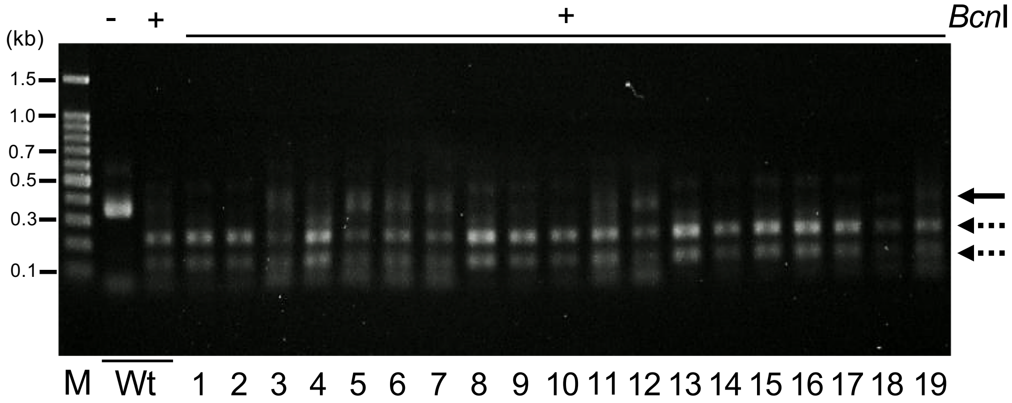

Supplementary Figure S2. CAPS analysis of *TaGASR7* locus in meristematic tissue. Genomic DNA was isolated from the meristematic tissue of wild-type (Wt) and GFP-positive embryos (nos.1-19) 3 days after plant bombardment and then subjected to a cleaved amplified polymorphic sequences (CAPS) assay. M, marker; –, undigested PCR products; +, *Bcn*I-digested PCR products. Black and dashed arrowheads indicate the positions of uncut and cut PCR products, respectively. The full-length gel image of Figure 1a is presented.

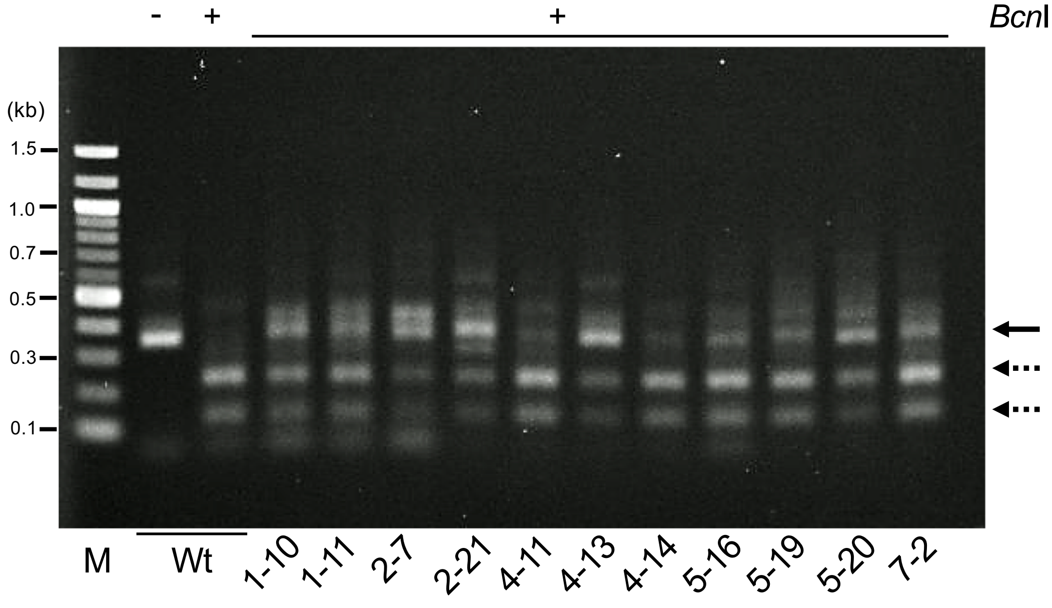

Supplementary Figure S3. CAPS analysis of *TaGASR7* locus in T_0 plants.

Genomic DNA was isolated from each fifth leaf of 11 bombarded plants and one wild-type (Wt) plant and then subjected to PCR and subsequent *Bcn*I restriction enzyme digestion. M, marker; –, undigested PCR products; +, *Bcn*I-digested PCR products. Black and dashed arrowheads indicate the positions of uncut and cut PCR products, respectively. The full-length gel image of Figure 2 is presented.

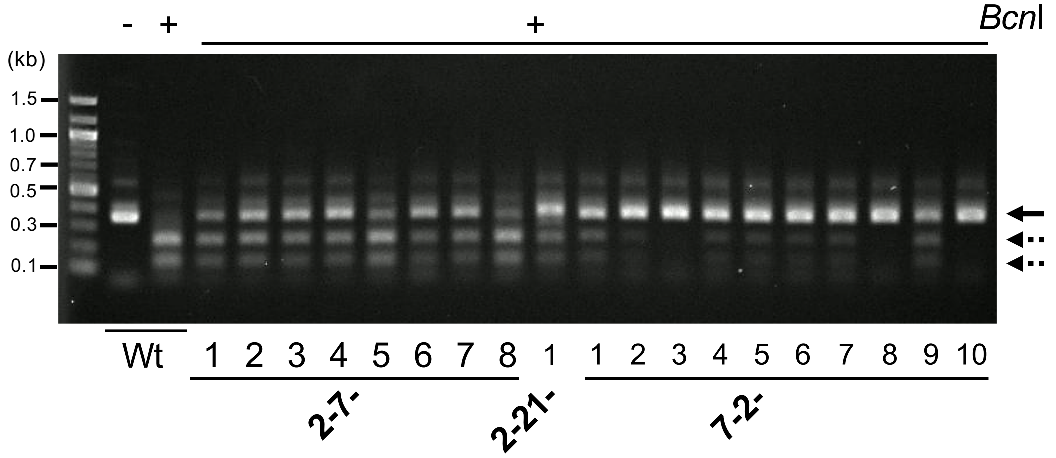

Supplementary Figure S4. CAPS analysis of *TaGASR7* locus in T_1 plants.

Genomic DNA isolated from each first leaf of independent T_1 plants derived from three T_0 mutants (2-7, 2-21, and 7-2) and one wild-type (Wt) plant. The DNA was subjected to PCR with *TaGASR7-A1, -B1* and *-D1* conserved primer sets. PCR products were digested with *Bcn*I restriction enzyme. −, undigested PCR products; +, *Bcn*I-digested PCR products. Solid and dashed arrows indicate the positions of uncut and cut PCR products, respectively. The Full-length gel image of Figure 3a is presented.

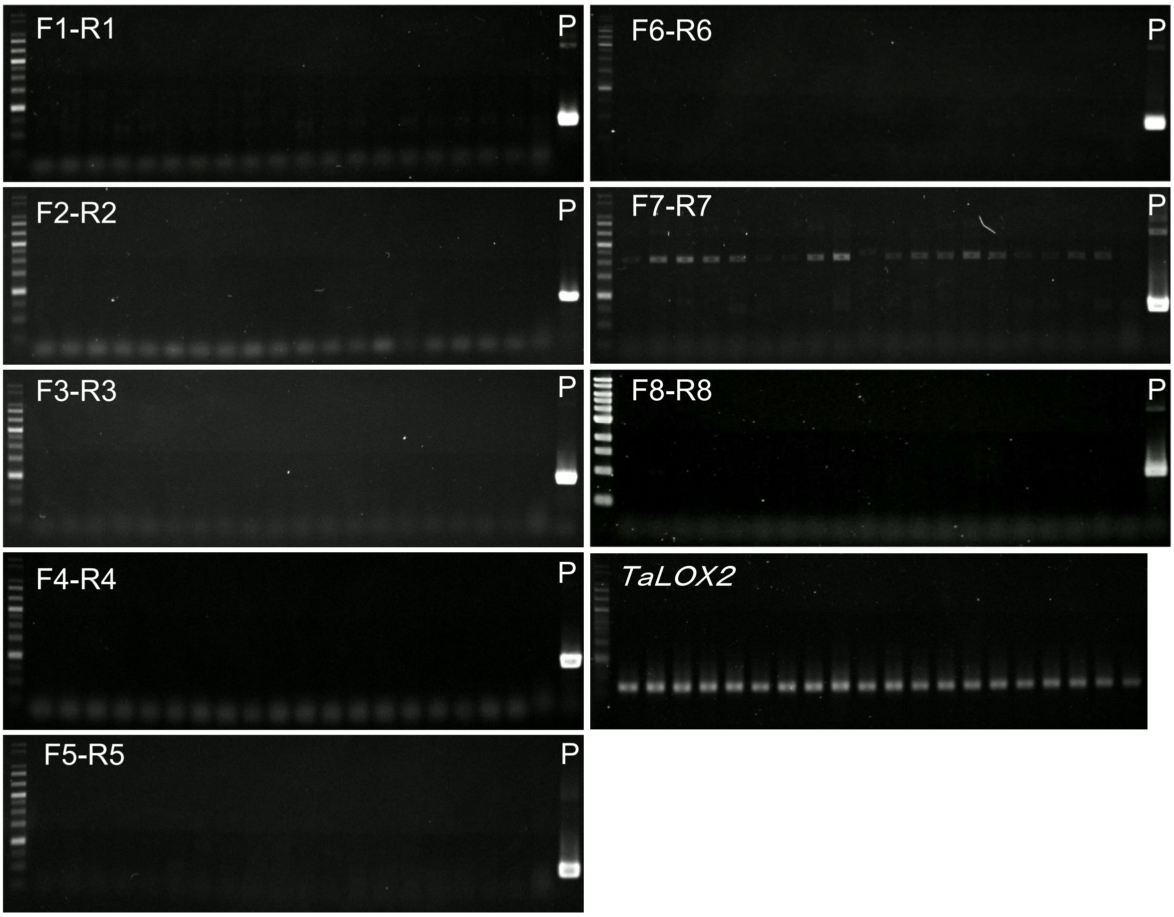

Supplementary Figure S5. Detection of Detection of vector DNA sequences in T1 mutant lines. Genomic polymerase chain reaction (PCR) analysis of T1 mutants (2-7-1~8, 2-21-1, 7-2-1~10) and wild-type (Wt) plants. Genomic DNA was extracted from each first leaf. Each of the three vectors was used as a positive control (P). The full-length gel image of Figure 4 is presented.

Supplementary Table 1

ID	Genotype	Pattern of mutation					
		A genome		B genome		D genome	
T_1-2-7-1	A A B b D d	Wt	Wt	Wt	+ T	Wt	+ T
T_1-2-7-2	A A B b d d	Wt	Wt	Wt	+ T	+ T	+ T
T_1-2-7-3	A A B b d d	Wt	Wt	Wt	+ T	+ T	+ T
T_1-2-7-4	A A b b D d	Wt	Wt	+ T	+ T	Wt	+ T
T_1-2-7-5	A A B B D d	Wt	Wt	Wt	Wt	Wt	+ T
T_1-2-7-6	A A b b D d	Wt	Wt	+ T	+ T	Wt	+ T
T_1-2-7-7	A A b b D d	Wt	Wt	+ T	+ T	Wt	+ T
T_1-2-7-8	A A B B D d	Wt	Wt	Wt	Wt	Wt	+ T
T_1-2-21-1	A a B b D d	Wt	+ T	Wt	+ A	Wt	- 35 bp
T_1-7-2-1	A A b b d d	Wt	Wt	+ A	+ A	+ A	+ A
T_1-7-2-2	A a b b d d	Wt	+ T	+ GT	+GT	+ A	+ A
T_1-7-2-3	a a b b d d	+ T	+ T	+ A	+ A	+ A	+ A
T_1-7-2-4	A a b b d d	Wt	+ T	+ A	+ A	+ A	+ A
T_1-7-2-5	A a b b d d	Wt	+ T	+ A	+ A	+ A	+ A
T_1-7-2-6	A a b b d d	Wt	+ T	+ A	+ GT	+ A	+ A
T_1-7-2-7	A a b b d d	Wt	+ T	+ A	+ A	+ A	+ A
T_1-7-2-8	a a b b d d	+ T	+ T	+ GT	+ GT	+ A	+ A
T_1-7-2-9	A a B b D d	Wt	+ T	WT	+ A	Wt	+ A
T_1-7-2-10	a a b b d d	+ T	+ T	+ A	+ GT	+ A	+ A

Supplementary Table 2

Primer name	primer sequence (5' →3')	Product size (bp)	Detection
TaGASR7 conserved F<br>*TaGASR7* conserved R	CTCAAGCCATGGGGTAAACCCACT<br>AACCGCAAGCTGGCACCCCAGAAG	364, 354, 342	*TaGASR7*<br>*-A1, B1, D1*
GASR7-A1/B1/D1-F<br>GASR7-A1-R	CCTTCATCCTTCAGCCATGCAT<br>CCACTAAATGCCTATCACATACG	560	*TaGASR7-A1*
GASR7-A1/B1/D1-F<br>GASR7-B1-R	CCTTCATCCTTCAGCCATGCAT<br>AGGGCAATTCACATGCCACTGAT	569	*TaGASR7-B1*
GASR7-A1/B1/D1-F<br>GASR7-D1-R	CCTTCATCCTTCAGCCATGCAT<br>CCTCCATTTTTCCACATCTTAGTCC	578	*TaGASR7-D1*
F1<br>R1	CTGCAGTGCAGCGTGACCCG<br>GACGGCGTTTAACAGGCTGGC	598	ZmPubi
F2<br>R2	CTGCTACCTCCAAGAGATCTTCTCC<br>GACTGGTCGAAGAAGATCTCCTTGT	828	*Cas9*
F3<br>R3	CATGACCAACTTCGACAAGAACCTC<br>GTAGAGGTAGAGCTTCTCGTTCTGG	961	*Cas9*
F4<br>R4	CATGAACACCAAGTACGACGAGAAC<br>AGGTAGAGGAAGTTCACGTACTTGG	900	*Cas9*
F5<br>R5	GATGCGCCAGAGTTGTTTCT<br>TCCGACTCGTCCAACATCAA	522	kanR<br>(aminoglycoside 3'-phosphotransferase coding gene)
F6<br>R6	TTAAGACCAAGCCCGTTATTCTG<br>GACTCTAGAGCGGATCCACAATA	625	TaU6-sgRNA
F7<br>R7	TTAGCCCTGCCTTCATACGC<br>ACCATGTGATCGCGCTTCT	763	*GFP*
F8<br>R8	GTATCCGCTCATGAGACAATAACC<br>TGGTCATGAGATTATCAAAAAGGA	1025	ampR<br>(β-lactamase coding gene)
TaLOX2 F<br>TaLOX2 R	CTCTGCCCGACGATCTGATCAAG<br>GGAACTGGTTGGTGATGGTGAGG	568	*TaLOX2*
